# Preparation and Application of Volatilized Wormwood Essence Derived Naturally into Green Insecticide

**DOI:** 10.3390/molecules29122877

**Published:** 2024-06-17

**Authors:** Shaoming Jin, Yaonan Wang, Tongtong Liu, Xiao Ning, Ruiqiang Liang, Kang Hu, Jin Cao

**Affiliations:** 1 National Institute for Food and Drug Control, Beijing 100050, China; 2 School of Pharmaceutical Sciences, Capital Medical University, Beijing 100069, China

**Keywords:** wormwood, essential oil, green pesticide

## Abstract

Naturally occurring substances and their derivatives function as vital resources for pesticides that can be used in fields, such as insecticide production and fungicide development. As a botanical entity displaying multifaceted biological functions, wormwood has received thorough scrutiny across multiple sectors. The insect repellency potency combined with antibacterial and antifungal activities of wormwood position it as a potential candidate for prospective development into eco-friendly chemical pesticides. In this research, Wormwood essential oil was procured via ethanol water under ultrasonic scenarios and subsequently diluted with PEG 400 to formulate green chemical pesticides. The defensive efficacy of this green pesticide on plants was validated through 2 weeks of clustered plant growth experiments. Active constituents that exerted their effects were scrutinized by GC-MS. Furthermore, this green pesticide also displays efficacious effects on the prevention and management of aphids, exhibiting a dose-dependent relationship. 4-terpenol, eucalyptol, carvacrol, and L-borneol were identified by GC-MS as the predominant active constituents in this green chemical pesticide. Wormwood can be leveraged to develop green chemical pesticides, which can protect plants without contaminating the environment.

## 1. Introduction

The incorporation of insecticides for assuring the normal growth of plants holds significant implications for agricultural production; however, some inappropriate misuse can also engender certain environmental intricacies [1,2,3]. Eco-friendly and environmentally sound insecticides will be advanced and utilized moving forward. Substances derived from plants will serve as a source of green insecticides with the potential to be endorsed for commercial applications [4,5]. Wormwood *(Artemisia argyi H. Lév. & Vaniot)* is a perennial herbaceous plant of the *Asteraceae* family, belonging to the Artemisia genus. Sometimes, it is also referred to as mugwort [6]. It has a prominent thick and long main root and many lateral roots. The underground rhizome is recumbent, and the stem is solitary. The plant has a strong aroma. The leaves are covered with gray-white short pubescent hairs, and there are white glandular and small concave spots [7]. The head inflorescence is elliptical, sessile or nearly sessile, and the achenes are oval or elongated. *Artemisia argyi* has a wide natural distribution, covering various parts of the world, including China, Russia, Mongolia, North Korea, Japan, etc. It mostly grows in low-altitude to medium-altitude areas such as wastelands, roadsides, riversides, and mountain slopes [8]. As a perennial herbaceous plant, wormwood is commonly used in alcoholic beverages such as absinthe. This bitter plant has long been used to alleviate pain and swelling and treat digestive problems, intestinal worms, and skin infections [9,10]. Its dry leaves are often picked before the flowers bloom in summer, impurities are removed, dried in the sun, and used as a medicinal herb. In China, they are commonly used for vomiting blood, bleeding, metrorrhagia, excessive menstruation, fetal bleeding, low abdominal pain, irregular menstrual cold, infertility due to a cold uterus, and can be used externally to treat skin itching [11,12].

Artemisinin is a compound found in wormwood and is believed to have useful anti-inflammatory effects [13]. Studies have shown that it works by antagonizing interleukin-12 and tumor necrosis factor α to treat inflammation by waiting for inflammatory factors [14]. Therefore, wormwood can help alleviate inflammatory symptoms such as pain, redness, fever, and swelling and is also used to treat labor pain, premenstrual pain, and joint and muscle pain [5]. Many studies on the anti-inflammatory effects of wormwood have focused on its treatment for patients with osteoarthritis and rheumatoid arthritis [15,16]. In addition, wormwood can treat serious gastrointestinal diseases caused by parasites such as pinworms, roundworms, and tapeworms. From a historical perspective, wormwood was once considered the preferred drug for treating intestinal worms, but due to the serious side effects associated with absinthe, it is no longer popular [17,18,19,20,21,22,23]. In a 2017 research report, extracts of *Artemisia absinthium* were found to be effective in killing *Hymenolepis nana* in laboratory mice. Although researchers have reported that mugwort extract is not as effective as the commonly used drug praziquantel to treat worms, considering the increasing resistance of *Hymenolepis nana* to parasitic agents such as praziquantel and albendazole, this discovery was considered important [24]. Wormwood has also been proven to have effective antibacterial and antifungal activities [25,26]. Wormwood has been proven to have anti-Staphylococcus aureus activity, which is one of the main causes of skin and soft tissue infections [27].

The volatile components can be extracted from the leaves and flowers of *Artemisia argyi* plants through steam distillation, forming common essential oils with high medicinal value [28,29]. Wormwood essential oil has a particularly strong odor, with a warm and sometimes bitter herbal aroma [30]. Wormwood essential oil has complex chemical components, containing over 75 different compounds [31,32]. The main ingredients include anisaldehyde, anisole, anisone, eucalyptus, camphor, and pinene. These compounds contribute to the therapeutic properties of oil and can be used as effective natural therapies for a range of health conditions. Artemisia essential oil is also known for its insect repellent ability, making it a popular choice for natural insect repellents. Its strong odor can effectively drive away mosquitoes, flies, and other pests [33].

In daily greenhouse and household farming, due to certain temperature and humidity conditions, common fungal and insect infestations such as powdery mildew and aphids occur frequently. Exploring effective biological pest control methods derived from natural plants is a green and organic approach. Powdery mildew mainly comes from fungi, such as *Sphaerotheca panosa*, *Erysiphe cichoracearum*, *Oidiuml eucoconium*, *Oidium euonymi japonicae*, etc. After being infected with these fungi, the leaves will turn yellow and wither, and the fungi will be transmitted through air flow, causing more plant infections. The expansion of the main white spot area indicates the development of the infection. Based on the long-term use of wormwood and its characteristics of sterilization and pest killing, and according to the initial screening findings, this study tried to use the environmentally friendly dilution of wormwood essential oil to spray infected plants to effectively control plant powdery mildew and aphids in the greenhouse and avoid causing damage to the environment.

## 2. Results and Discussion

### 2.1. Insecticidal Test Results

During the 2-week experimental period, the infected parts of the plants were evenly sprayed with a spray bottle, each time spraying 50 mL of distilled water or a diluted solution of essential oil extract. In both observation subjects, the uninfected plant group did not develop any related diseases. The uninfected plants placed in the same mesh with the infected plants did not show any transfection between the plants corresponding to powdery mildew due to indoor cultivation, but the corresponding plants of aphid disease had a small portion of aphids transferred to the original uninfected plants. In the calculations, the transferred aphids were also merged and counted according to independent units of the same yarn net.

#### 2.1.1. Anti Powdery Mildew Effect

**Figure 1:**
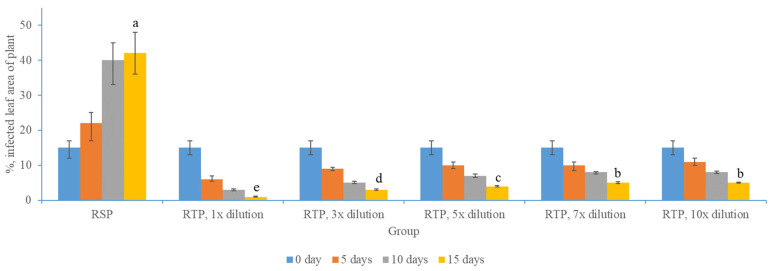
The anti-infection effect of different diluted wormwood essential oil. The data are shown as the proportion of infected areas on leaves. There was a significant statistical difference in the data between the RSP and RTP groups after 15 days, and the statistical differences among different dilution treatments within the group were also significant. The symbols a b c d e in the illustration signify statistical significance, with identical symbols suggesting no statistical disparity, while dissimilar symbols reveal statistical variations.

In this research, the infection severity was evaluated according to the area covered by white hyphae [34]. The experiment result showed that the plants of the RB1 and RB2 groups did not suffer from powdery mildew infection under independent indoor planting, indicating effective plant protection and limited air circulation in the space, without causing related fungal contamination. However, during the observation of the RSP group, white spots expanded during the 2-week cultivation period, indicating the survival and diffusion of related fungi in the infected area. The areas stained with white spots were classified according to the leaf area, and there was about two to three times expansion, effectively recording the development of related diseases. At the same time, after 2 weeks, the infected leaves of the plant group showed yellowing. For the RTP group sprayed with diluted wormwood essential oil extract, corresponding white spot control was achieved. Even at a 10-fold dilution, the leaves produced less yellowing, indicating that wormwood essential oil extract has a certain fungal killing and inhibitory effect. Please refer to the display in Figure 1 for details.

As shown in Figure 1, the proportion of damaged blades in the RSP group reached almost 42% after two weeks, and the results of each treatment group showed that, under a diluted solution of about 10 times, there was a significant reduction and killing effect on powdery mildew, and the proportion of damaged blades was 5%. The higher the concentration, the stronger the killing effect. Interestingly, after seven times or more, it tended to stabilize, indicating a balance between disease expansion and the killing effect of the relevant effective substances. However, in terms of leaf health, it was shown that the dilution solution had a protective effect on plants. It was discovered that powdery mildew caused early leaf senescence by diminishing photosynthesis [35]. This occurs primarily due to hyphal coverage diminishing the functional leaf area, thereby reducing the assimilation rate of the remaining leaf area and affecting the gas exchange [36]. The essential oil of Artemisia and its diluted solution efficiently curbed diseased leaf areas and restored infected areas, demonstrating its capacity to eradicate infected fungi and enhance the plant’s recovery from damage.

#### 2.1.2. The Effectiveness of Aphid Prevention and Control

**Figure 2:**
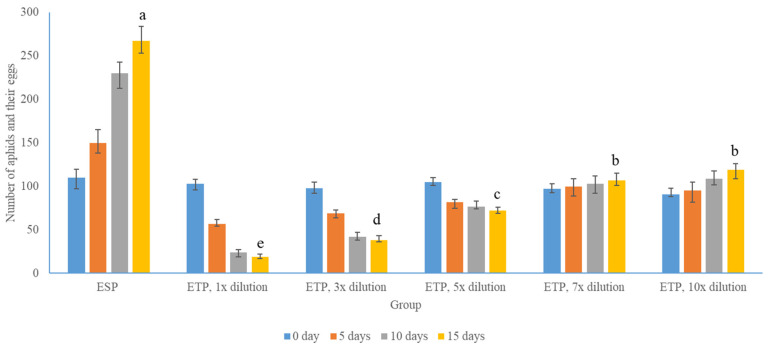
The aphid prevention and control effect of different diluted wormwood essential oil. The data are shown as the number of aphids and their eggs. There was a significant statistical difference in the data between the ESP and ETP groups after 15 days, and statistical differences among different dilution treatments within the group were also significant. The symbols a b c d e in the illustration signify statistical significance, with identical symbols suggesting no statistical disparity, while dissimilar symbols reveal statistical variations.

Aphids, as a major pest of indoor plants, can cause yellowing and wilting of leaves due to their puncture and absorption of nutrients from the leaves [37]. As sap-feeding insect nuisances, they inflict economic damage on crops. They possess advanced adaptive mechanisms, including the utilization of detoxification enzymes in defensive host plant interactions [38,39]. During the 2-week experimental period, EB1, as a healthy control, was not affected by aphids, while EB2 in the same gauze as the related infected plants was affected to varying degrees by aphids. This indicates that, within a certain range, eradicating aphids and their eggs can reduce aphid infestation, and aphids can infect different plants. As an observation control group, the ESP group was completely invaded by aphids, with half of its leaves turning yellow or wilting, and to some extent, the number of aphids (including eggs) increased significantly. The number was recorded in the corresponding group. The treatment group ETP using the coefficient solution of *Artemisia argyi* essential oil extract showed varying degrees of hindrance to the growth of aphids due to the actions of relevant drug solutions. However, when the dilution ratio was too large, the solution diluted more than seven times in the experiment may be due to a decrease in the concentration of relevant effective substances. After the cultivation period, the number of aphids (including eggs) involved was 1.1 and 1.3 times the original level, indicating a weak inhibitory effect on aphids. However, coefficients below five times had a more significant inhibitory effect, with values of 0.2, 0.4, and 0.7 times the original level at one, three, and five times the dilution, respectively. This indicates that the extract of *Artemisia argyi* essential oil and its diluent have a certain degree of aphid killing effect. In addition, the growth and health status of the leaves can also indicate the related reduction effect. Dilutions below 5 times can significantly control the yellowing rate of the leaves, while diluents at 7 and 10 times can also reduce the proportion of yellowing in the leaves, but it is not significant. The results can be seen in Figure 2.

As shown in Figure 2, wormwood essential oil had good efficacy in killing aphids, and the number of aphids (including eggs) in the ESP group was 267 after 2 weeks, while this number in the ETP group was 19. Also, there was a clear concentration correlation, and the effect of wormwood essential oil extract was only within a certain effective concentration range, which had a reducing effect on aphids. At the same time, in terms of leaf health, although there was a protective effect at a dilution of more than seven times, it was not significant, reflecting the relationship between concentration and effect mentioned above. The number of aphids and eggs was counted from one group containing three plants, due to aphids laying eggs very quickly, and during the 2-week experimental observation period, eggs continuously developed into aphids [40,41]. For the convenience of data collection and analysis, we counted the number of adults and eggs on infected plants at the same time every day to examine the effectiveness of the green insecticide we prepared. Despite no distinction between insect eggs and adults, the number of insect eggs in the plant group exposed to undiluted essential oil was macroscopically lower than the diluted treatment group, signifying our green pesticide impacted aphid reproduction to a certain extent.

### 2.2. Determination of Biomarkers and Estimation of Quality Specifications

The extract of wormwood essential oil was mainly composed of volatile substances; among which, the main components that could kill bacteria and insects were 4-terpenol, eucalyptol, carvacrol, L-borneol, etc. The antibacterial and insecticidal effects were related to the concentrations of the above components to a certain extent. Therefore, as a comprehensive quality indicator for the development of this type of natural pesticide, the above components were determined.

#### 2.2.1. Detection Results

**Figure 3:**
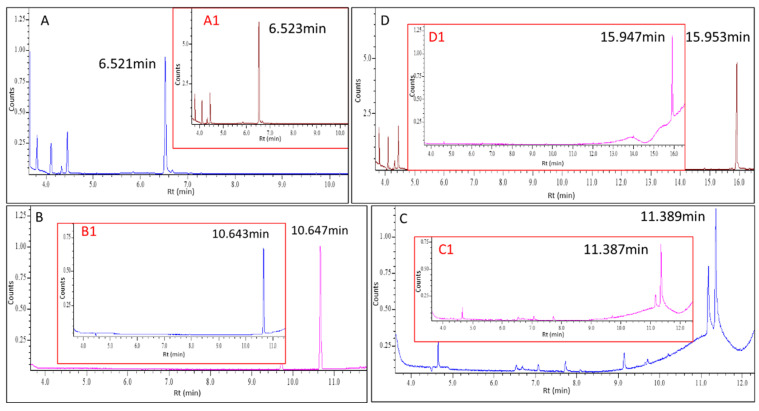
The extract ion chromatogram (EIC) of wormwood essential oil extracts (shown in (**A**–**D**)) and four standard reference materials (shown in A1/B1/C1/D1).

In an endeavor to accurately identify the active components in wormwood essential oil, gas chromatography-mass spectrometry (GC-MS) was utilized. GC-MS is the most commonly used instrument for analyzing essential oil components [42,43,44]. Simultaneously, four standard reference materials were subjected to identical analysis under these stringent conditions, and their outcomes were meticulously compared to those of the mugwort essential oil, as illustrated in Figure 3.

The retention time and charge to the mass ratio serve as the basis to distinguish compounds. With consistency in these two parameters, it is generally assumed that the substances are identical [45,46,47]. As shown in Figure 3, the signals of the four standard reference substances match well with the signals of the extract of wormwood oil: the retention time of carvacrol was 6.523 min and 6.521 min, the retention time of 4-terpenol was 10.643 min and 10.647 min, the retention time of L-borneol was 11.387 min and 11.389 min, and the retention time of eucalyptol was 15.947 min and 15.953 min. These findings attested to the fact that wormwood essential oil comprised these four efficacious substances.

#### 2.2.2. Quality Specification Evaluation

According to the experimental results, the dilution exhibited bactericidal and insecticidal effects only when 4-terpenol, eucalyptol, carvacrol, and L-borneol reached a certain concentration. However, due to the impact of different batches of wormwood leaves on the yield of essential oil extracts, as a potential source of pesticides, we further analyzed the preparation effects of 10 batches of wormwood leaf extracts and found that 4-terpene alcohol, eucalyptol, carvacrol, and L-borneol can maintain effective concentrations within a certain concentration range (334.3~458.5 μg/L) through corresponding extraction and dilution steps. Considering the results of multiple batches of wormwood leaves, their quality specifications can be guaranteed. Based on actual trials of indoor plants, it has also been demonstrated that wormwood leaves can serve as a candidate source of effective pesticides for the sterilization and pest control of greenhouse plants.

### 2.3. Experimental Optimization

#### 2.3.1. Extraction Optimization of Essential Oil Substances

In the extraction of essential oils, there are often various methods such as steam distillation, soaking extraction, and supercritical fluid extraction. Experiments have compared the methods of steam distillation, soaking extraction, ultrasonic extraction, and redissolution involved in this study. Among them, the essential oil obtained by steam distillation is relatively pure, with a high concentration of effective substances, but its preparation rate is not high, only below 0.3% mass concentration. In addition, various substances in the same batch of wormwood leaves have significant differences in substance concentration in multiple replicates, which may be due to the influence of distillation temperature, time, etc. In common soaking extraction, olive oil or ethanol is often used for soaking and heating extraction to obtain more effective parts. However, due to the high number of impurities and the dark color of the solution, it is not conducive to later dilution applications. In this study, ultrasonic extraction using ethanol water was used, and after concentration and redissolution, the obtained raw solution had a lighter color, good fluidity, and the effective substances met the requirements. Therefore, the above treatment method was determined.

#### 2.3.2. Confirmation of Effective Insecticidal Substances

The active substances within the essential oil extracts from different extraction methods exhibited considerable disparity. After multiple iterations of extraction, it was found that 4-terpenol, eucalyptol, carvacrol, and L-borneol were the main active ingredients in essential oil extracts, which can be stably obtained in multiple batches of extracts. Following the rigorous comparison between the application methods of steam distillation and this controlled experimentation, the efficacious concentration indicators of the four substances were more precisely defined and refined, and the respective proportional relationships were also validated through actual plant insecticidal investigations.

#### 2.3.3. Optimization of the Formulation of Insecticidal Essential Oils

When optimizing the extraction method to obtain insecticidal essential oils, it is imperative to assess their conformity with spraying stipulations and the maintenance of active compounds on plants during genuine application to validate the potency of insecticidal effects. To increase the adhesion of essential oil solution on leaves, the results of different diluents were compared. The experiment utilized polyethylene glycol 400 (PEG 400), pectin, and cellulose for scrutiny. Cellulose, due to the presentation of particle attachment following drying, influenced the appearance of the plant leaf surface and was not favorable for leaf growth. Conversely, pectin possesses certain advantages in solubility; however, when juxtaposed with PEG 400, its fluidity introduced certain complexities, which was not optimal for spraying implementation. Therefore, PEG 400 was eventually deployed to enhance the adhesive attributes of the solution. The experimentation demonstrated robust adherence and superior fluidity within the 2–15% concentration range. Ultimately, a concentration ratio of 10% of the original solution was determined for deployment.

## 3. Materials and Methods

### 3.1. Instruments and Reagents

A 7890A/7000C triple quadrupole gas chromatography-mass spectrometer (Agilent Technology Co., Ltd., Beijing, China), a DB-5MS GC column (30 m × 0.25 mm × 0.25 μm, Agilent Technology Co., Ltd., Beijing, China), a XP205 analytical balance (1 in 100,000), an AL204 analytical balance (1 in 10,000) (Mettler, Greifensee, Switzerland), a XHF-D high-speed disperser (Ningbo Xinzhi Biotechnology Co., Ltd., Ningbo, China), and a CF 16RX II centrifuge (Hitachi Corporation, Tokyo, Japan) were used.

4-terpenol, eucalyptol, carvacrol, and L-borneol were purchased from Beijing Manhage Biotechnology Co., Ltd. (Beijing, China), with purity greater than 98%. Methanol and acetonitrile were chromatographically pure (Thermo Fisher Company, Waltham, MA, USA). Anhydrous NaSO_4_ was analytically pure and purchased from Sinopharm Group Chemical Reagent Co., Ltd., Shanghai, China. Fresh and dry wormwood leaves were purchased from the medicinal market, and *Rosa gallica* L. and *Epipremnum aureum (Linden* & *André)* were domesticated. In this experiment, fine mesh was used for isolation to ensure that relevant bacteria and pests did not leak out.

### 3.2. Preparation of Wormwood Essential Oil Extract

First, 200 g of fresh and dried wormwood leaves were blended into powder, then 300 mL of 80% ethanol solution was added and sonicated for approximately 30 min. Following this, centrifugation at 8000 rpm was essential, and the upper layer of the solution was filtered using qualitative filter paper. Subsequently, 100 mL of the solution was concentrated to 10 mL of viscous solution. This was dispersed with 20 mL of 95% ethanol and then filtered to procure a light green solution. Given its substantial concentration of ethanol, there was a propensity for evaporation that could cause dehydration. Therefore, it was diluted at a ratio of 1:30 with 50% ethanol, alongside polyethylene glycol 400 (PEG 400), for a 10% PEG 400 solution, finally yielding the raw extract of wormwood essential oil for testing. During the comparative examination, anhydrous water was employed to dilute the original solution in multiple dilutions (namely 1×, 3×, 5×, 7×, and 10×) on a regular basis. This procedure served to scrutinize and optimize the concentration of the primary active compound.

### 3.3. Determination of Effective Substances in the Wormwood Essential Oil Extract

#### 3.3.1. Preparation of Standard Reference Solution

To formulate a blend of 10 mg each of 4-terpenol, eucalyptol, carvacrol, and L-borneol (to 0.01 mg), each substance was dissolved in 10 mL acetonitrile, yielding a standard stock solution of 1 mg/mL. These stock solutions were utilized for preparing a single standard or mixed standard after diluting and mixing.

#### 3.3.2. Sample Processing

Then, 1 mL of the sample solution was taken and incorporated with 10 mL of acetonitrile and vortexed thoroughly. Ultrasonic extraction was conducted for 10 min, followed by the addition of anhydrous magnesium sulfate to dehydrate. The solution was centrifuged at 10,000 rpm for 5 min to isolate the supernatant, which was filtered through a 0.22 µm GHP membrane and reserved for analysis.

#### 3.3.3. GC-MS Conditions

According to the conventional operating procedures of essential oil analysis, the conditions for GC-MS were determined [48]. A DB-5ms column was used for chromatographic separation; the sample injection volume was 1 µL; the fractional flow ratio was set to 15:1; the carrier gas was helium; the flow rate was set to 1 mL/min; the temperature of the injection port was set to 280 °C; and the heating up procedure was set as follows: the initial temperature was 60 °C, maintained for 2 min, then increased to 100 °C at 10 °C/min, maintained for 2 min, then increased to 220 °C at 15 °C/min, maintained for 2 min, and finally increased to 280 °C at 20 °C/min and maintained for 4 min. The GC-MS interface and ionization source temperature were 280 °C and 230 °C, respectively. The solvent delay time was 4 min.

### 3.4. Comparative Trials of Hothouse Plants

The study was performed indoors, utilizing infected and non-infected *Rosa gallica* and *Epipremnum aureum* as controls. Three groups were established: non-infected, infected control, and infected medication. The infected medication was subjected to five distinct diluted release concentrations.

#### 3.4.1. Powdery Mildew

Powdery mildew caused by *Sphaerotheca aphanis* infection was chosen as the research object of the resistance and control trial [49,50]. *Rosa gallica*, a common landscape plant, was selected for the examination of the efficacy of wormwood essential oil against powdery mildew [51]. The plants were grown in pots, and they were divided into different groups based on their infection status, each group containing three plants. RB1 and RB2 contained uninfected plants and served as blank control groups. Plants infected with white spots were evenly divided into six groups according to the leaf infection area. One group was used as the positive control (RSP), and distilled water was sprayed during planting, while the other five groups were used as the treatment groups (RTP). RB1 was placed separately by gauze mesh; RB2 was placed in the same mesh with infected plants (RSP group); and the five treated groups (RTP 1×, 3×, 5×, 7×, and 10×) were placed separately during the two-week experimental period. During the overall 2-week cultivation period, distilled water or essential oil extract diluents were uniformly sprayed twice a day. We recorded the status daily, calculated based on the surface area of the blades.

#### 3.4.2. Aphid Disease

*Aphis spiraecola*, also known as green citrus aphid, was the research object of the aphid resistance experiment in this study. It is widely distributed in China and commonly found on various plants [52,53]. *Epipremnum aureum*, a common indoor plant used for purifying air, was selected for the examination of the efficacy of wormwood essential oil against aphids [54,55]. Plants without aphids and insect eggs were used as blank controls and placed separately (EB1) and in the same mesh as related infected plants (EB2). Plants infected with aphids and insect eggs were evenly divided into six groups with three plants in each group: one group was used as the control (ESP), and distilled water was sprayed during planting, while the other five groups were used as the treatment groups (ETP), spraying the extract of *Artemisia argyi* essential oil in 5 dilution concentrations (1, 3, 5, 7, and 10 times the original concentration), and each group was isolated independently with a mesh. At the same time, a barrier was set at the bottom of the plant to allow aphids to fall to the roots without cross-infection between different groups. During the overall 2-week cultivation period, we sprayed twice a day with distilled water or diluted essential oil extract. Their status was recorded daily, calculated based on the total number of aphids and insect eggs.

#### 3.4.3. Statistical Analysis

Statistical analysis was executed using SPSS 11.0 (IBM, Armonk, NY, USA) for Windows, and data were shown as the mean ± SD in the figures. Data were analyzed via Student’s *t*-test (comparing two samples) or ANOVA (comparing more than two samples), with multiple pairwise comparisons being carried out using Duncan’s multiple range test.

## 4. Conclusions

As a natural plant, the rich efficacy of wormwood indicates its potential to develop into a natural source of green chemical pesticides. The results of this study indicate that wormwood essential oil acquired by ultrasonic extraction using ethanol water, containing active ingredients such as 4-terpenol, eucalyptol, carvacrol, and L-borneol, has been proven to have anti-powdery mildew abilities and aphid prevention and control effects in different plant growth experiments. It only took two weeks to achieve good results and control the development of plant infections and insect pests. As a high-performance diluent, PEG 400 can evenly spray the diluted wormwood essential oil on the surface of leaves, ensuring the long-term attachment of effective insecticides. As an environmentally friendly pesticide derived from plants, its effectiveness against pests and diseases alone is insufficient. Future research will assess its influence on plants to ensure normal growth is unaffected. This study offers a novel approach for the development of green chemical pesticides from natural sources.

## Data Availability

All data are included in this manuscript.
